# The iron-sensing aconitase B binds its own mRNA to prevent sRNA-induced mRNA cleavage

**DOI:** 10.1093/nar/gku649

**Published:** 2014-08-04

**Authors:** Julie-Anna M. Benjamin, Eric Massé

**Affiliations:** Department of Biochemistry, RNA Group, University of Sherbrooke, 3201 Jean Mignault Street, Sherbrooke, Quebec J1E 4K8, Canada

## Abstract

Aconitase is an iron–sulfur protein and a major enzyme of the TCA cycle that catalyzes the conversion of citrate to isocitrate under iron-rich conditions. In *Escherichia coli*, aconitase B (AcnB) is a typical moonlighting protein that can switch to its apo form (apo-AcnB) which favors binding its own mRNA 3′UTR and stabilize it when intracellular iron become scarce. The small regulatory RNA (sRNA) RyhB has previously been shown to promote RNase E-dependent degradation of *acnB* mRNA when it was expressed from an ectopic arabinose-dependent promoter, independently of intracellular iron levels. In marked contrast, we report here that expression of RyhB under low-iron conditions did not result in *acnB* mRNA degradation even when RyhB was bound to *acnB* ribosome binding site (RBS). Genetic and biochemical evidence suggested that, under low-iron conditions, apo-AcnB bound to *acnB* 3′UTR close to a RNase E cleavage site that is essential for RyhB-induced *acnB* mRNA degradation. Whereas RyhB can block *acnB* translation initiation, RNase E-dependent degradation of *acnB* was prevented by apo-AcnB binding close to the cleavage site. This previously uncharacterized regulation suggests an intricate post-transcriptional mechanism that represses protein expression while insuring mRNA stability.

## INTRODUCTION

Aconitases are iron–sulfur proteins and major enzymes of the tricarboxylic acid (TCA) cycle that catalyze the reversible conversion of citrate to isocitrate. The bacterium *Escherichia coli* possesses two isoforms of aconitases, AcnA and AcnB, whose expression varies according to environmental conditions. For instance, AcnA is expressed during stress and stationary phase whereas AcnB is the major TCA enzyme expressed during exponential growth under aerobic conditions (1). However, AcnB is unstable under certain conditions of environmental stress. For example, AcnB contains a 4Fe–4S group that participates in catalysis but this essential structure becomes disassembled when cells are exposed to oxidative stress or low intracellular iron and that results in loss of activity (2–4).

AcnB is not only sensitive to environmental conditions but it also acts as a moonlighting or bifunctional protein. Whereas AcnB functions as a catalytic enzyme under iron-rich conditions in *E. coli*, it becomes a regulatory RNA-binding protein when intracellular iron becomes scarce (apo-AcnB). Indeed, under low-iron conditions AcnB can bind its own mRNA 3′UTR, which increases *acnB* mRNA stability (2–3,5). This creates a dynamic equilibrium between both AcnB states (enzyme/RNA-binding), which depend on the intracellular iron pool (4). This dual function was first described in higher organisms where it was shown that cytoplasmic apo-aconitase could bind mRNAs (6,7). Subsequent investigations in eukaryotes revealed that aconitase mRNAs carried iron-responsive elements (IREs) motifs (8,9). Oxidative stress and iron starvation induce aconitases expression to maintain iron homeostasis, thus classifying aconitases as iron-regulatory proteins (IRPs) (10,11). Acn-IRPs can bind to IREs located in the 5′ or 3′ end of mRNA UTRs to either protect mRNAs from degradation or to prevent translation, respectively. IREs are also found in the 3′UTR of the transferrin receptor mRNA (*trf1*), where they induce mRNA stabilization by protecting mRNA from cleavage by an uncharacterized endonuclease (12). In contrast, IREs found in the 5′UTR of ferritin lead to translational inhibition of the Fe-storage protein, similarly to many others Fe-metabolism genes (13,14).

The small regulatory RNA (sRNA) RyhB that is strongly expressed in response to low-iron condition has been reported to be an additional factor of regulation of *acnB* mRNA in *E. coli* (15). Small RNAs (sRNAs) in bacteria are important regulators of cellular functions by modulating gene expression in response to changes in the environment. More than 80 sRNAs have been identified so far in *E. coli* (16). A majority of these sRNAs regulate mRNAs by direct base-pairing, which affect either positively or negatively their translation and stability (17,18). In many cases, sRNAs require the RNA chaperone Hfq for optimal regulation by promoting sRNA–mRNA pairing and by stabilizing sRNAs against degradation *in vivo* (19,20). The chaperone Hfq can also act as a direct translational repressor by competing with initiating 30S ribosomal subunit for accessibility to AU-rich sequences in the vicinity of the ribosome binding site (RBS) (21,22). Such AU-rich sequences have been shown to act as translational enhancers by increasing interactions with ribosomal protein S1 and by stabilizing mRNA (23–28).

RyhB is one of the most extensively studied Hfq-dependent sRNAs. When cells grow under iron-rich conditions, the transcriptional regulator Fur (Fe^2+^-Fur) represses *ryhB* promoter. In contrast, Fur becomes inactive and relieves repression of *ryhB* under conditions of iron starvation (15,29). When iron is depleted, RyhB represses ∼20 different mRNAs, which encode iron-dependent proteins. When bound to those mRNAs, RyhB shuts down translation and promotes their degradation through recruitment of the RNA degradosome, a protein complex that includes endoribonuclease E (RNase E), a 3′-5′ polynucleotide phosphorylase (PNPase), an RNA helicase (RhlB) and an enolase (15,22,30–33). By regulating iron-using proteins, RyhB contributes actively to increase the levels of free intracellular Fe^2+^ (iron sparing) during iron starvation by reducing the expression of iron-dependent proteins (34).

Previous work from our group has shown that RyhB represses *acnB* mRNA under defined conditions (31). Here, we extend these observations to show the importance of intracellular iron levels in determining the outcome of RyhB-induced *acnB* repression. When *E. coli* is grown under high-iron conditions, RyhB expression results in *acnB* mRNA degradation. In contrast, low-iron conditions favor translational repression of *acnB*, leaving *acnB* mRNA intact. Our data suggest that apo-AcnB protects *acnB* mRNA from RNase E-mediated cleavage after RyhB pairing. This mechanism highlights a new level of regulation of sRNA-induced mRNA degradation that is linked to the metabolic status of *E. coli*, which influences the enzyme/RNA-binding properties of AcnB.

## MATERIALS AND METHODS

### Strains and plasmids

Strains and plasmids used in this study are listed in Supplementary Table S1. The methods used to obtain all constructions are described in Supplemental experimental procedures. Derivatives of *E. coli* MG1655 were used in all experiments. *Escherichia coli* expression strain BL21 (DE3) was used for overproduction and purification of AcnB_3xFLAG_ protein. DH5α strain was used for routine cloning procedures. Cells were grown at 37°C in Luria-Bertani (LB) medium with addition of antibiotics depending on plasmids used. In the case of cells carrying pFRΔ, pRS1551, pNM12, pBAD24 and pET-21b, ampicillin (final concentration, 50 μg/ml) was added. Cells harboring pGD3 and pBAD33 plasmids were grown in the presence of chloramphenicol (final concentration, 30 μg/ml).

### β-Galactosidase assays

Kinetics assays for β-galactosidase activity were performed as described (35) using a SpectraMax 250 microtitre plate reader (Molecular Devices, Sunnyvale, CA, USA). Briefly, overnight bacterial culture incubated at 37°C were diluted 1000-fold in 50 ml of fresh LB medium and grown under mechanical shaking at 37°C. Fe-starvation was induced by addition of a final concentration of 200 μM of the Fe chelator Dip when cultures reached an OD_600_ of 0.1. The formula *V*_max_/OD_600_ was used to calculate specific β-galactosidase activity. Results reported in this study correspond to a minimum of three independent experiments.

### Western blot analysis

Cells were grown in LB medium at 37°C. Appropriate inducer (arabinose or Dip) was added to cells at an OD_600_ of 0.1. Proteins were harvested by precipitation with trichloroacetic acid (final concentration, 1% (w/v)) as described (36). Proteins were resuspended in protein loading gel electrophoresis buffer before loading on sodium dodecyl sulfate-polyacrylamide gel electrophoresis (SDS-PAGE) (7% acrylamide) and transferred on a nitrocellulose membrane. An anti-AcnB polyclonal antibody was used at a dilution of 1:5000 (kind gift of Dr Jeffrey E. Green, National Cancer Institute). The IRDye 800CW-conjugated goat anti-rabbit secondary antibody (Li-Cor Biosciences, Lincoln, NE, USA) was used at a dilution of 1:15 000 and revealed on an Odyssey Infrared Imaging System (Li-Cor Biosciences). Quantifications were carried out using the Odyssey Application Software.

### RNA extraction and northern blot analysis

Total RNA was extracted using the hot phenol procedure (37). Cells were grown to an OD_600_ of 0.5 and 0.1% arabinose or 200 μM of Dip were added. Determination of RNA half-life was performed by addition of 250 μg/ml rifampicin to the culture before total RNA extraction. In the case of *acnB* northern blots, 40 μg of total RNA were loaded on an agarose gel (1% agarose in 3-(N-Morpholino)propanesulfonic acid (MOPS) buffer). In the case of RyhB sRNA and *sodB*_130_-*acnB*_+67_ construct northern blots, 5 and 15 μg, respectively of total RNA were loaded on 4% polyacrylamide gel (acrylamide 29:1, 8 M urea). After migration, the RNA was transferred by capillarity (agarose gel) or electro-transferred (acrylamide gel) to a Hybond-XL membrane (Amersham Biosciences) and UV crosslinked. Prehybridization, hybridization and washes were performed as described (36). Membranes were exposed to phosphor storage screens and analyzed using a Typhoon Trio (GE Healthcare) instrument. Quantification was performed using the ImageQuant software (Molecular Dynamics).

### RNA immunoprecipitation

Cells carrying plasmids pBAD24-*acnB* and pBAD24-*acnB-3xFLAG* (see Supplemental Material for details) were grown in LB medium to an OD_600_ of 0.1, at which point both AcnB_3xFLAG_ and AcnB were induced by adding arabinose (final concentration of 0.1%). When the culture reached an OD_600_ of 0.5, Dip (200 μM final) was added to induce RyhB for 10 min. The culture was then chilled on ice for 20 min to stop growth. Cells were then centrifuged and suspended in binding buffer (20 mM Tris–HCl, 0.25 M KCl, 5 mM MgCl_2_, 10% glycerol, 0.1% Tween 20, pH 8.0) (1 ml). After another centrifugation, cells were suspended in 2 ml of binding buffer and lysed using a French press (800 psi, three times). The lysate was then cleared by centrifugation (17 000 × *g*, 30 min, 4°C) and proteins were quantified. At this point, 20 μl of soluble fraction were mixed with the same volume of protein sample buffer and 80 μl of the same fraction were used for total RNA extraction (input). The remaining soluble fraction (0.8 ml) was incubated with Anti-FLAG M2 Affinity gel (Sigma-Aldrich, St Louis, MO, USA) for 2 h on a rotating platform (all steps performed at 4°C). The suspension was centrifuged and the beads were washed twice with Tris-buffered saline (TBS) (500 μl each). The beads were then suspended in TBS (100 μl) and FLAG-peptide (Sigma-Aldrich, 15 μg) was added. Eluted RNAs were then subjected to phenol–chloroform extraction, followed by precipitation (2.5 vol. ethanol supplemented by 20 μg of glycogen) of the aqueous phase. Proteins were isolated from the organic phase by acetone precipitation. RNA samples were analyzed by quantitative Real-Time PCR (qRT-PCR) (for *acnB*_2749_, *acnB*_2749-Mstem_, *acnB*_2749-stem_, *acnB*_+20-3′UTR_ constructs) or by northern blot (endogenous *acnB*). qRT-PCR reactions were performed four times on a Rotor-Gene 3000 device (Corbett Life Science, Concord, NSW) with Platinum SYBR Green qPCR SuperMix-UDG (Invitrogen) (29). Protein samples were analyzed by western blots.

### *In vitro* RNA synthesis and radiolabeling

Oligonucleotides used to generate DNA template for *in vitro* transcription are listed in Supplementary Table S3. T7 RNA polymerase (Roche Diagnostics GmbH, Mannheim, Germany) was used for radiolabeled probe transcription for northern blot analysis. Antisense transcript of genes of genes of interest were generated with a published method (36). A published protocol (22) was used for transcription of RNA used in secondary structure probing, RNase E degradation assays and toeprinting assays. ^32^P-pCp 3′ end radiolabeling was carried out as described (33) and 5′-^32^P-end radiolabeling were done as published (36).

### RNA degradosome degradation assays

Experimental procedure for RNase E cleavage sites characterization on ^32^P-pCp 3′ end radiolabeled RNA transcripts has been performed as described (33). RNase E-FLAG was purified as published (33).

### Aconitase B purification

Purified AcnB_3xFLAG_ were prepared as described (33), with some modifications. *Escherichia coli* (BL21 [DE3] pLysS/pET21b-*acnB-3xFLAG*) was grown in LB medium (2 l) containing ampicillin (50 μg/ml) and chloramphenicol (30 μg/ml) at 30°C, until it reached an OD_600_ between 0.5 and 0.6. During growth, Dip was added (final concentration of 200 μM) when the culture had reached an OD_600_ of 0.1. Expression of AcnB_3xFLAG_ was induced with Isopropyl β-D-1-thiogalactopyranoside (IPTG) (1 mM) for 3 h at 30°C. An additional fast protein liquid chromatography (FPLC) exclusion purification (G200) was performed using modified Ip buffer (Tris–HCl 50 mM, NaCl 150 mM, glycerol 5% and Ethylenediaminetetraacetic acid (EDTA) 1 mM, pH 7.4). Purified apo-AcnB_3xFLAG_ was used in all relevant experiments.

## RESULTS

### RyhB-induced nucleolytic cleavage of *acnB* mRNA depends on intracellular Fe levels

**Figure 1.**
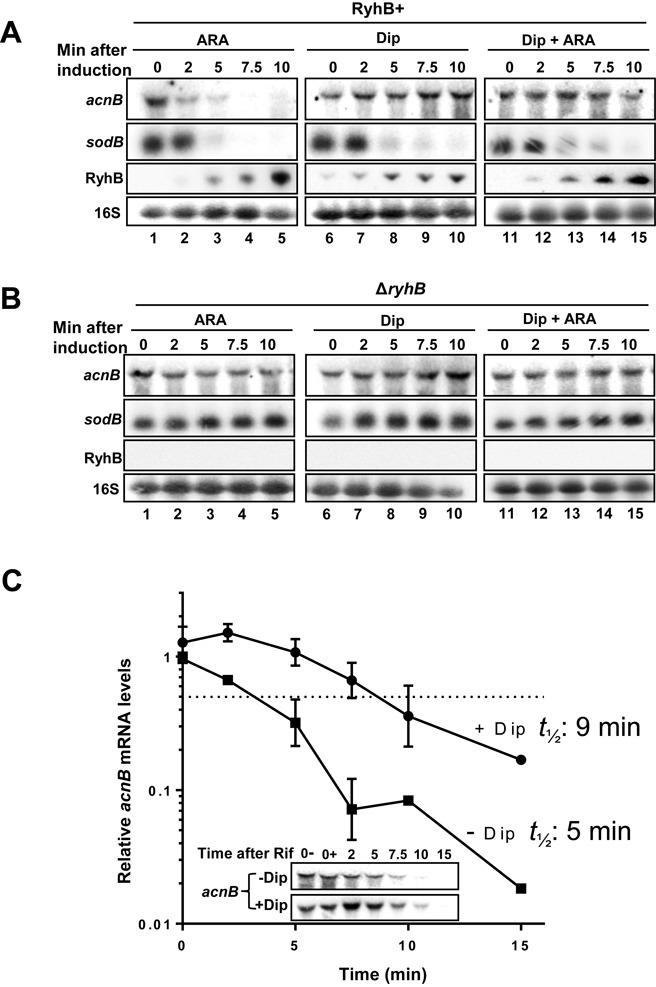
The mRNA *acnB* is post-transcriptionally regulated as a function of Fe status. (**A**) Northern blots analysis of RyhB and Fe starvation effects on *acnB* mRNA levels. Total RNA extracted from strains EM1455 (Δ*ryhB*), carrying pBAD-*ryhB*, and EM1055 (WT) strains was hybridized with an *acnB*-specific probe. Cells were grown to an OD_600_ of 0.5 in LB medium at which point arabinose (0.05% final) (strain EM1455 carrying pBAD-*ryhB*, lanes 1–5), 2.2′-dipyridyl (Dip) (200 μM final) (strain EM1055, lanes 6–10) or both (strain 1455 carrying pBAD-*ryhB*, lanes 11–15) were added at time 0. Total RNA was extracted at the indicated time. The well-characterized RyhB target *sodB* mRNA is shown as a positive control for RyhB-induced degradation under all conditions. 16S rRNA was used as a loading control. (**B**) Same results as in panel (A) but total RNA extracted from strains EM1455 (Δ*ryhB*) carrying pNM12 and EM1238 (Δ*ryhB*) in which there was no RyhB expression. (**C**) Decay rates of *acnB* mRNA in LB medium. Fe chelator Dip (200 μM) was added at time −10 min. Then, rifampicin (Rif) was added (250 μg/ml final concentration) at time 0 before total RNAs were extracted at the indicated time points. These data are representative of four independent biological replicates quantified by densitometry and normalized to time 0 (0+) for both +Dip and −Dip. Mean and SD values experiments are shown. The dashed line corresponds to 50% decrease in mRNA levels. Inset: Northern blots using *acnB* probe showing *acnB* mRNA stability. Calculated *acnB* half-life value is indicated beside respective experimental conditions on the graph.

The *acnB* mRNA has been previously shown to be down-regulated by the sRNA RyhB when expressed from the arabinose-inducible promoter of the pBAD-*ryhB* construct (31). In these experiments, RyhB was induced independently of intracellular iron status and Fur activity, both of which normally control the expression of the endogenous *ryhB* promoter (38,39). Notably, AcnB has been shown to shift from catalytic (holo-AcnB) to RNA-binding regulatory mode (apo-AcnB) under conditions of iron starvation (4,5). Therefore, we asked whether *acnB* mRNA could be protected from RyhB-induced mRNA degradation when AcnB switched to binding its own mRNA (apo-form) under conditions of low Fe. We first addressed this question by inducing expression of RyhB under two different modes of induction. The first mode involved cultures in Fe-rich medium (Figure 1A, left panels) of EM1455 (Δ*ryhB*) cells carrying an arabinose-inducible pBAD-*ryhB* plasmid (0.1% arabinose, Ara). The second mode involved EM1055 (WT) cells grown in Fe-poor medium by adding the iron chelator 2,2′-dipyridyl (Dip) and where RyhB is under the control of its endogenous promoter. Results showed that when RyhB was expressed in cells carrying the pBAD-*ryhB* plasmid in Fe-rich medium, it triggered a rapid degradation (within 5 min) of *acnB* mRNA (Figure 1A, lanes 1–5). The mRNA *sodB* was used as a positive control in these experiments and showed the expected degradation kinetics of a RyhB target (≤5 min). These results were in agreement with our previous observations that RyhB-induced degradation of *acnB* when expressed under the control of pBAD-*ryhB* when arabinose was present in the medium (31). In marked contrast, when RyhB sRNA was induced from its endogenous promoter under low Fe conditions, *acnB* mRNA remained stable even after 10 min of RyhB expression. This result was unexpected given that the fact that RyhB-sensitive *sodB* mRNA became fully degraded under similar conditions (Figure 1A, lanes 6–10). These results suggested that decreased intracellular iron levels may help to stabilize *acnB* mRNA although RyhB sRNA was fully expressed and able to induce degradation of control target *sodB* mRNA. To confirm that these results (Figure 1A, lanes 1–5) were not due to an arabinose-related effect, a third mode of induction was investigated. In this case, EM1455 cells carrying pBAD-*ryhB* were grown under condition of iron depletion (Dip, 250 μM) and were treated with arabinose to induce RyhB. Induction of RyhB under low iron showed that *acnB* mRNA remained stable throughout the experiment, although RyhB expression induced *sodB* degradation within 5 min (Figure 1A, lanes 11–15). These results confirmed that iron-depleted conditions protected *acnB* mRNA against RyhB-induced degradation.

Control experiments performed in the absence of RyhB (Δ*ryhB*) showed no effect on both *acnB* and *sodB* mRNAs (Figure 1B, lanes 1–5). Unexpectedly, addition of Dip (250 μM) induced a 2-fold increase of *acnB* mRNA levels when cells had been treated with the iron chelator for 10 min (Figure 1B, lanes 6–10). This *acnB* mRNA stabilization effect was similar in the case of another Fe chelator, diethylene triamine pentaacetic acid (DTPA), which unlike the Fe chelator Dip is not internalized by cells (Supplementary Figure S1A). These results were consistent with the interpretation that DTPA favored the switch to apo-AcnB, which would then bind its own mRNA (4). Taken together, our finding suggested that *acnB* mRNA was stabilized against RyhB-induced mRNA degradation in a Fe-depleted medium.

To determine whether the promoter of *acnB* was sensitive to intracellular iron levels, we measured promoter activity in the presence or absence of Dip. Activities of *acnB* promoter showed that there were no significant differences between these two experimental conditions (Supplementary Figure S1B). This result was in contrast to that observed for the *ryhB* promoter in which case there was a strong response to iron depletion (Supplementary Figure S1B, *ryhB*). A possible explanation to account for the fact that the stability of *acnB* mRNA increased under depleted iron (Figure 1A and B, compare lane 6 with lane 10) was that AcnB switched to its apo form favoring its binding to its own mRNA and increasing its stability. This possibility was tested by measuring *acnB* mRNA half-life in extracts of Δ*ryhB* cells grown in the presence or absence of Dip, for 15 min. As expected, *acnB* mRNA stability was significantly increased in the presence of Dip, from 5 to 9 min as compared to growth in the absence of Dip (Figure 1C). These data were consistent with the interpretation of post-transcriptional stabilization of *acnB* under iron-depleted conditions.

### Apo-AcnB binds 3′UTR of *acnB* mRNA under conditions of iron depletion

**Figure 2.**
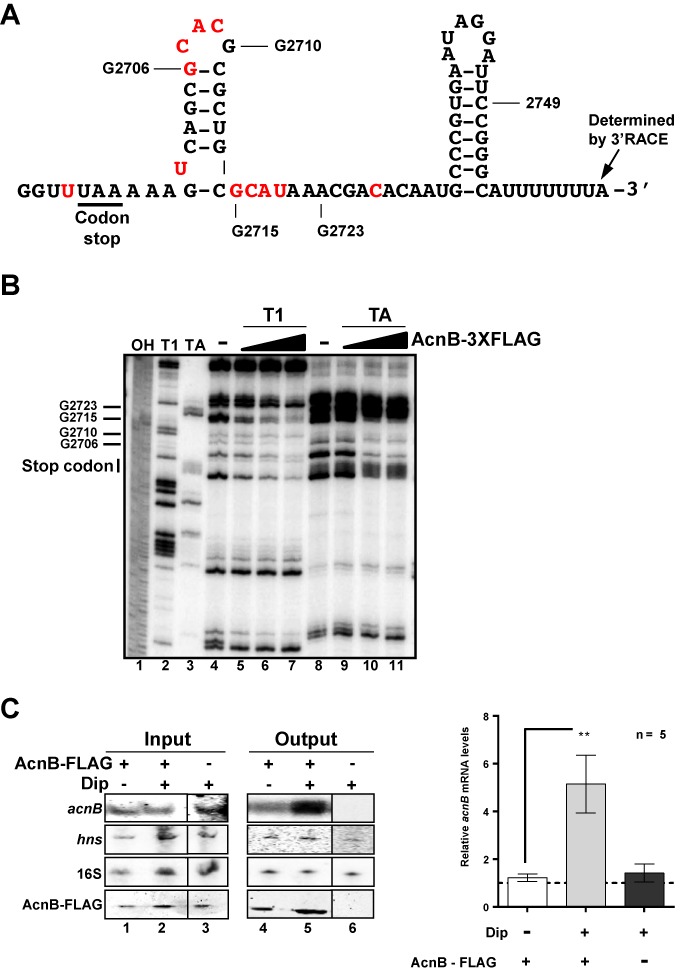
AcnB_3xFLAG_ binds to *acnB* RNA 3′UTR *in vitro* and *in vivo* in Fe-depleted medium. (**A**) Secondary structure and sequence of the 3′UTR of *acnB* mRNA. In red, nt protected by apo-AcnB in footprint experiment (see panel B). (**B**) Footprint analysis of purified AcnB_3xFLAG_ protein showing binding site on *acnB* 3′UTR RNA. (Lane 1) NaOH ladder. (Lane 2) RNase T1 ladder. (Lane 3) RNase TA ladder. (Lanes 4–7) *acnB* RNA T1 digestion with addition of increasing amounts of purified AcnB_3xFLAG_ protein (ratio *acnB* RNA:AcnB_3xFLAG_, 1:0, 1:1, 1:2 and 1:10, respectively). (Lanes 8–11) TA digestion of *acnB* RNA with addition of increasing amounts of AcnB_3x FLAG_ protein (same ratio as used for T1 digestion). (**C**) AcnB_3xFLAG_ binds to *acnB* mRNA in Fe-depleted medium *in vivo*. RNA IP performed with pBAD-*acnB*_3xFLAG_ (JAB146A) (lanes 1, 2, 4 and 5) compared to pBAD-*acnB* (JAB151) (lanes 3 and 6). Dip (200 μM) was added 10 min before stopping cells growth and proceeding to RNA IP. Northern blots, hybridized with an *acnB* RNA probe and western blot, using an anti-AcnB antibody, were performed on samples before (input) and after (output) RNA IP. 16S rRNA and *hns* mRNA were used as negative controls for AcnB_3xFLAG_ enrichment. Northern blot signals were quantified by densitometry and normalized to *acnB* mRNA levels without Dip. Mean and SD values from five biological replicates are shown. Statistical one-way ANOVA test significance is shown by asterisks (*P* < 0.002).

Previous *in vitro* experiments have suggested that apo-AcnB bound to the 3′UTR of *acnB* mRNA (5). In these experiments, the authors used a 308 nucleotide-long sequence covering the 3′UTR of *acnB* without giving experimental evidence for the 3′ end of the transcript. We set up experiments to define the 3′ end of *acnB* by performing 3′RACE experiments, which indicated that the transcript terminated 67 nt after *acnB* ORF (Figure 2A, sequence and structure of *acnB* 3′UTR). Next, we performed experiments to identify the apo-AcnB binding site on *acnB* 3′UTR. Footprinting assays in which radiolabeled *acnB* 3′UTR was subjected to partial cleavage by RNase T1 (cleaves single-stranded Gs) and RNase TA (cleaves single-stranded As) in the presence of increasing amounts of purified AcnB_3xFLAG_ protein were performed. We found clear protection of the structure surrounding the stem–loop located in 3′UTR (Figure 2B, lanes 4–11). These results suggested that apo-AcnB bound to the 3′UTR of *acnB* mRNA in the stem–loop structure immediately downstream of the ORF (red nucleotides in Figure 2A).

Because previous results suggested AcnB bound to *acnB* 3′UTR *in vitro*, we next performed AcnB_3xFLAG_ RNA immunoprecipitation (RIP) experiment to determine whether AcnB could bind to *acnB* mRNA under conditions of iron depletion. Cells were grown in LB medium to an OD of 0.5 after which point cells were left untreated or Dip was added for 15 min. Cells were then lysed and lysate was applied to a FLAG-binding column. After washes and elution, *acnB* mRNA was detected by northern blot analysis. Densitometry analysis revealed a significant enrichment (4- to 6-fold) in *acnB* mRNA recovery in the presence of Dip as compared to its absence (Figure 2C, lanes 4 and 5). Control experiments were performed by monitoring *acnB* mRNA recovery in a strain carrying plasmid pBAD24-*acnB* without FLAG. Under these control conditions, there was no recovery of *acnB* mRNA from cultures grown under Fe depletion (Figure 2C, lanes 3 and 6). Furthermore, there were no variations between the various conditions of culture with the non-target control *hns* mRNA and 16S ribosomal RNA (Figure 2, lanes 1–6). These results gave further support to the interpretation that interaction between AcnB_3xFLAG_ and *acnB* mRNA depended on depleted intracellular Fe.

### Implication of *acnB* 3′UTR in regulation of its mRNA stability

**Figure 3.**
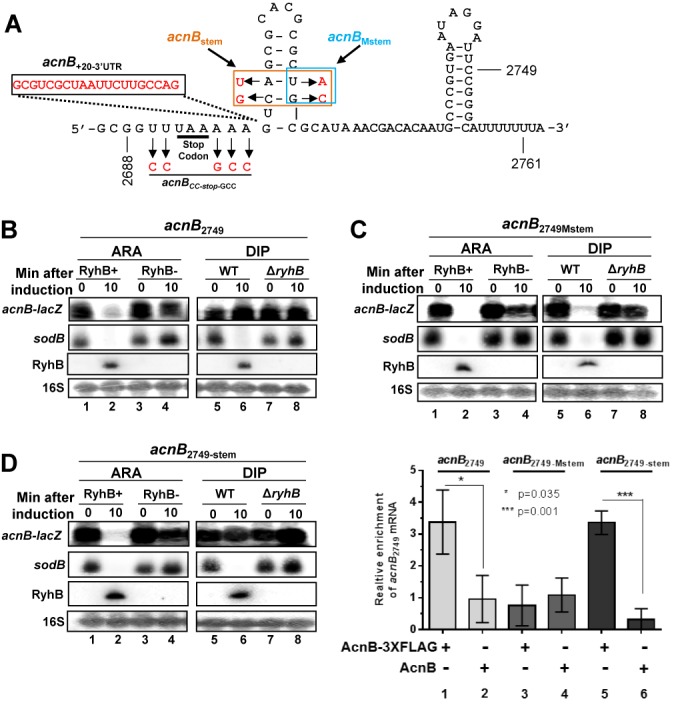
Apo-AcnB binding on *acnB* mRNA is essential for prevention of sRNA-induced degradation. (**A**) Scheme of *acnB* 3′UTR showing *acnB*_2688_, *acnB*_2749_, *acnB*_2749-Mstem_, *acnB*_+20-3’UTR_ and *acnB*_CC-stop-GCC_ transcriptional *lacZ* fusions constructs. The end of three commonly used transcriptional *acnB*’-*lacZ* fusion length 2688, 2749 and 2761 are annotated on the scheme. Letters in red corresponds to mutated nt for the indicated construct. (**B**) Northern blot of *acnB*_2749_ transcriptional *lacZ* fusion, *sodB* mRNA, RyhB sRNA and 16S rRNA that were hybridized with the corresponding RNA probes (ARA: strain KP1135 carrying pGD3-*ryhB* (RyhB+) or empty pGD3 plasmid (RyhB−); Dip: strains EM1055 (WT) or EM1238 (Δ*ryhB*)). Total RNA extraction was performed at the indicated time (0 or 10 min), at an OD_600_ of 0.5. Northern blots showing the effect of RyhB on *acnB*_2749_ (B), *acnB*_2749-Mstem_ (**C**) and *acnB*_2749-stem_ (**D**) transcriptional *lacZ* fusions. Expression of RyhB was induced at time 0 by addition of arabinose (0.05% final) or Dip (200 μM final). (**E**) AcnB_3xFLAG_ RNA-IP on *acnB*_2749_, on *acnB*_2749-Mstem_ and on *acnB*_2749-stem_ constructs were expressed from pBAD33-derived plasmids in JAB292 cells (*acnB*736::*kan*). AcnB protein induction (OD_600_ of 0.1) was performed from pBAD-*acnB*_3xFLAG_ and from pBAD24 plasmids. Then, when OD_600_ reached 0.5, RNA IP was performed. qRT-PCR analysis was performed with a probe covering the *acnB* 3′UTR for determination of *acnB* constructs enrichment. Mean with SD values from triplicates experiments are shown. Statistical one-way ANOVA test significance is shown by asterisks.

Results described above (Figure 2) suggested that *acnB* 3′UTR mediated the stability of *acnB* mRNA through interaction with AcnB under conditions of iron starvation. To investigate the involvement of *acnB* 3′UTR in AcnB binding, we engineered a transcriptional *lacZ* fusion, which carried all but the last 12 nt of *acnB* mRNA (fused at nt 2749, see Figure 3A). The fusion *acnB*_2749_-*lacZ* reproduced the same pattern of degradation previously observed (Figure 1A and B) 10 min after RyhB expression under control of the Ara-inducible promoter (Fe-rich condition) (Figure 3B, lanes 1 and 2) or in the presence of Dip (Figure 3B, lanes 5 and 6). Furthermore, data indicated (Figure 2B) that the stem–loop located immediately downstream of *acnB* 3′UTR could bind to AcnB under iron depletion. To investigate the role of this stem–loop in *acnB* 3′UTR, we constructed an *acnB*_2749Mstem_-*lacZ* fusion that was mutated for 2 nt within the stem structure. This mutation disrupts the stem–loop secondary structure as suggested by Mfold (http://mfold.rit.albany.edu/?q=mfold. Data not shown). Northern blots indicated that *acnB*_2749Mstem_-*lacZ* was completely degraded after 10 min of RyhB expression whether RyhB was expressed from plasmid with Ara (Figure 3C, lanes 1 and 2) or, endogenously, in the presence of Dip (Figure 3C, lanes 5 and 6). To address the question whether the RNA sequence or the stem–loop structure was responsible for the loss of regulation, we constructed a fusion carrying compensatory mutations that reconstituted the stem–loop in *acnB* 3′UTR (*acnB*_2749-stem_, Figure 3A). Northern blots revealed that, under typical growth conditions, the *acnB*_2749-stem_ construct was degraded when RyhB was expressed from Ara (Figure 3D, lanes 1 and 2) but remained intact when RyhB was expressed in cells cultured in the presence of Dip (Figure 3D, lanes 5 and 6). These results were interpreted to suggest that the stem–loop structure located immediately downstream of *acnB* ORF played an important role in the stability of *acnB* mRNA when RyhB is expressed.

To investigate the possibility that AcnB bound *acnB* constructs with different affinities, depending on the 3′UTR sequences, we performed RNA IP assays. In these experiments, FLAG-tagged AcnB (AcnB_3xFLAG_) was used to quantitate binding affinity of the various constructs described above (Figure 3, *acnB*_2749_, *acnB*_2749Mstem_, *acnB*_2749-stem_). Cells were grown to mid-log phase, at which point they were treated with Dip (250 μM, 10 min), followed by lysis and purification (see ‘Materials and Methods’ section for details). Enrichment of the specific construct *acnB*_2749_ was then measured by qPCR. Results showed that transcriptional constructs *acnB*_2749_ and *acnB*_2749_-stem had comparable enrichment with AcnB_3xFLAG_ relative to untagged AcnB (Figure 3E, lanes 1 and 2, and lanes 5 and 6). In contrast, *acnB*_2749Mstem_ construct showed no enrichment with AcnB_3xFLAG_ (Figure 3E, lanes 3–4). These results established that the stem–loop structure was essential for AcnB binding to *acnB* 3′UTR under conditions of iron depletion.

### The 3′UTR of *acnB* mRNA is essential for RyhB-induced cleavage

**Figure 4.**
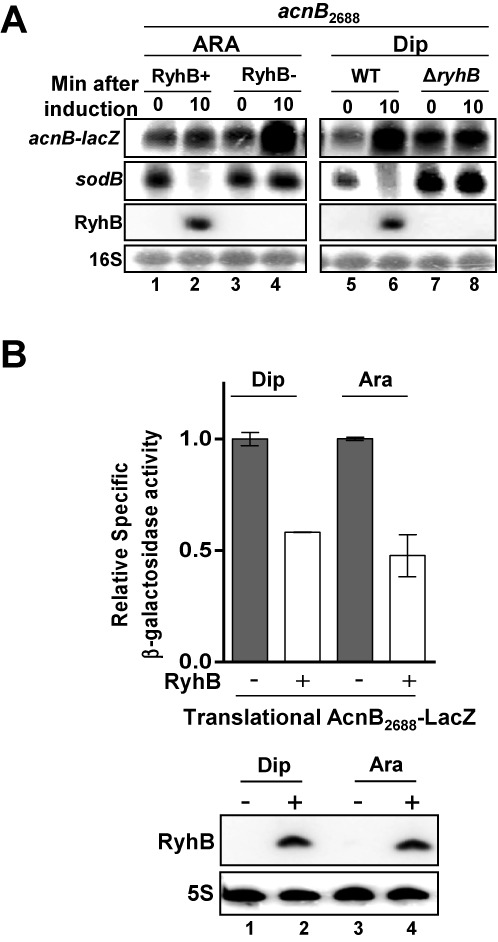
The *acnB* 3′ end is essential for RyhB-induced mRNA degradation. (**A**) Northern blot of total RNA of transcriptional *acnB*_2688_*-lacZ* fusion hybridized with *acnB* RNA probe. RNA was extracted after 10 min of RyhB induction with arabinose (0.05% final) or by Dip (200 μM final) (ARA: strain KP1135 carrying pGD3-*ryhB* (RyhB+) or empty pGD3 plasmid (RyhB−); Dip: strains EM1055 (WT) or EM1238 (Δ*ryhB*)). RyhB sRNA was hybridized to compare its expression in both conditions. 16S rRNA was used as loading control. (**B**) β-Galactosidase assays were performed with translational AcnB_2688_-LacZ fusion, which excluded *acnB* 3′UTR and its last two codons. Dip (200 μM final) for WT strain (EM1055) or arabinose (0.05% final) for pBAD-*ryhB* transformed strain (EM1455) were added to induce RyhB expression at an OD_600_ of 0.1. Specific β-galactosidase activity was measured in triplicates. Mean and SD of relative β-galactosidase activity (normalized to conditions without RyhB) are shown on graph. The empty vector pNM12 (EM1455) or Δ*ryhB* (EM1238) strains were used as a control for AcnB_2688_-LacZ without RyhB. Northern blot with RyhB probe on total RNA was performed at an OD_600_ of 0.5 along with β-galactosidase assays. 5S rRNA was used as loading control.

The data described above suggested that binding of AcnB to *acnB* 3′UTR interfered with RyhB-induced degradation of the mRNA. To add support to this interpretation, we constructed a transcriptional fusion (*acnB*_2688_-*lacZ*) comprising all but the last two codons of *acnB* ORF (Figure 3A). The reasoning behind the use of this construct was that it would allow RyhB binding to the translation initiation region (Supplementary Figures S2A, S2B and S2C) but would prevent apo-AcnB binding at the 3′UTR of *acnB* mRNA. The fusion *acnB*_2688_-*lacZ* was tested in cells grown under usual conditions (Figure 1A). Northern blots showed that *acnB*_2688_-*lacZ* remained resistant to the expression of RyhB after 10 min of induction (Figure 4A, lanes 1 and 2).

Although these observations suggested that RyhB expression could not induce *acnB*_2688_-*lacZ* degradation, they did not exclude the possibility that RyhB could nevertheless bind the construct. To confirm that RyhB was still able to bind *acnB*_2688_ transcript and block translation, we constructed a translational AcnB_2688_-LacZ fusion. Specific β-galactosidase activities indicated that RyhB significantly reduced expression of translation, regardless of the mode of induction (Dip, Ara) of RyhB (Figure 4B). These results, together with those shown in Figure 4A, strongly suggested that even if RyhB could bind *acnB* mRNA, the absence of the 3′UTR region prevented sRNA-induced mRNA cleavage. To further investigate the effect of RyhB pairing on 30S ribosomal subunit binding to *acnB* mRNA translation initiation region (TIR), we performed a toeprint assay in the presence of increasing amount of RyhB. Densitometry analysis revealed a strong inhibition (10-fold) of 30S subunit binding in the presence of added RyhB (Supplementary Figure S3, compare lanes 7 and 11, and 7 and 13). Moreover, the addition of purified Hfq chaperone contributed to block 30S subunit binding on *acnB* mRNA TIR by an additional 2.3-fold (Supplementary Figure S3, compare lanes 9 and 10, 11 and 12, 13 and 14). Control experiments showed that excess molar ratio of DsrA sRNA had no effect on 30S ribosomal subunit binding on *acnB* (Supplementary Figure S3, lane 15).

### Characterization of RNA degradosome RyhB-induced cleavage site on *acnB* mRNA

**Figure 5.**
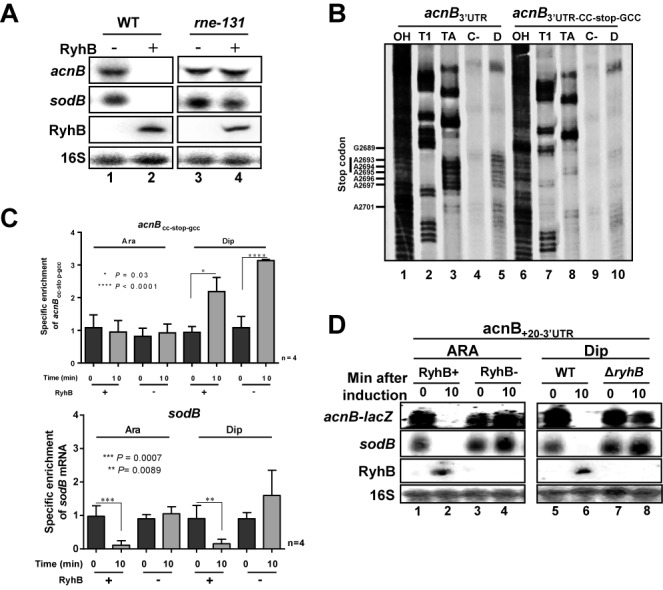
RyhB induces RNase E cleavage in *acnB* mRNA 3′ end. (**A**) Northern blot of *acnB* mRNA were performed on total RNA extracted from strain EM1455 and JF133 (*rne131* mutant). Both stains were transformed with plasmid allowing arabinose-dependent RyhB expression (pBAD-*ryhB*) or with control vector (pNM12). Cells were grown in LB medium to an OD_600_ of 0.5 and RyhB sRNA was induced at time 0 by addition of arabinose (0.01% final). Total RNA extraction was performed after 10 min of induction. (**B**) RNA degradosome degradation assay to map RNase E cleavage site on *acnB* 3′UTR. Effect of purified RNA degradosome (0.5 ng/μl final concentration) on ^32^P-pCp 3′ end labeling *acnB*-3′UTR (lanes 4 and 5) and *acnB*_3′UTR-CC-stop-GCC_ (lanes 9 and 10). (Lanes 1 and 6) NaOH ladder. (Lanes 2 and 7) RNase T1 ladder. (Lanes 3 and 8) RNase TA ladder. (Lanes 4 and 9) Radiolabeled *acnB* RNA alone (see Figure 3A for cleavage site map). (**C**) qRT-PCR of *acnB*_CC-stop-gcc_ transcriptional *lacZ* fusion from total RNA extraction. RyhB sRNA expression was induced by addition of arabinose (0.05% final) for pGD3-*ryhB* (KP1135) or by addition of Dip (200 μM final) for wild-type (EM1055). Total RNA extraction was performed at the indicated time, at mid-logarithmic growth phase. Endogenous *sodB* mRNA levels after RyhB expression are also shown. Mean and SD values of four replicates experiments are shown. Asterisks correspond to statistical significance from one-way ANOVA test. (**D**) Northern blot using *acnB* probe showing the effect of RyhB expression on *acnB*_+20-3′UTR_*-*transcriptional *lacZ* fusion (after 10 min). Before RNA extraction, RyhB sRNA expression was induced by addition of arabinose (0.05% final) for pGD3-*ryhB* (KP1135) or by addition of Dip (200 μM final) for wild-type (EM1055) at time 0. Empty plasmid pGD3 or Δ*ryhB* strains were used as negatives controls for ARA and Dip panels, respectively.

Most *trans*-acting sRNAs that induce degradation of their target mRNAs usually recruit RNase E through Hfq-mediated interaction (40). To confirm this mechanism in relationship to RyhB and *acnB*, we performed pulse-expression of RyhB in *rne131* background, which carried a deletion of RNase E C-terminus that prevented RNase E recruitment by Hfq. Northern blots confirmed that RNase E was essential for *acnB* mRNA degradation after a 10 min of RyhB induction with Ara (Figure 5A, lanes 2 and 4). The possibility that *acnB* 3′UTR carried a cleavage site recognized by RNase E (33) was investigated by degradation assays using purified RNA degradosome in the presence of radiolabeled *acnB* 3′UTR (from nt 2569 to nt 2767). Degradation assay revealed a strong cleavage signal near the stop codon of *acnB* ORF (Figure 5B, compare lanes 4 and 5), which mapped between nt 2690 and 2697 (see Figure 3A) close to *acnB* stop codon. We then mutagenized the cleavage site by modifying the sequence UUUAAAAA to CCUAAGCC (see Figure 3A). The mutant construct was then subjected to RNA degradosome assay. Results showed that CCUAAGCC mutations in the end of *acnB* ORF prevented cleavage by RNase E (Figure 5B, compare lanes 5 and 10). These results suggested that RNase E required the original AU-rich sequence near the stop codon of *acnB* mRNA to induce cleavage. To confirm that the RNase E-dependent cleavage site (Figure 5B) was essential, we mutated the *acnB*_2749_-*lacZ* construct to introduce the CCUAAGCC mutation that blocked RNase E cleavage. Cells harboring the construct *acnB*_2749CC-stop-GCC_ were subjected to RyhB expression and data analyzed by qRT-PCR. The *acnB*_2749CC-stop-GCC_ construct was completely resistant to RyhB-induced mRNA degradation under Fe-rich condition (Figure 5C, compare *acnB*_2749CC-stop-GCC_ and *sodB* panels, columns 1 and 2), highlighting the importance of the RyhB-induced cleavage site that mapped at the end of *acnB* ORF (Figure 5B, nt 2692–2697).

Next, we asked whether the 3′ end of *acnB* could function ectopically on *sodB*, a mRNA that is also a target of RyhB. We used a truncated version of *sodB* mRNA (*sodB*_130_) which has previously been shown to be resistant to RyhB-induced mRNA degradation (33). The construct was fused to the 3′ end of *acnB* to obtain *sodB*_130_*-acnB_+_*_67_ (Supplementary Figure S4A for details). As expected, this construct was sensitive to RyhB sRNA-dependent degradation under Fe-rich conditions (Supplementary Figure S4B, compare lanes 5 and 10). In contrast, induction of RyhB by Dip led to a slower degradation of the *sodB_130_-acnB_+_*_67_ construct, suggesting that *acnB* 3′ end can act ectopically to partially stabilize a particular mRNA from RyhB-induced degradation (Supplementary Figure S4).

Our results suggested that apo-AcnB protected *acnB* mRNA against RyhB-induced mRNA cleavage by occluding RNase E access to the cleavage site. This possibility required that the apo-AcnB binding site must remain proximal to the cleavage site for competitive inhibition. To address this possibility, we initially constructed a *lacZ* fusion in which a sequence of 20 nt was inserted between RyhB-induced RNase E cleavage site and apo-AcnB binding site (*acnB_+_*_20-3′UTR_-*lacZ*) (Figure 3A). It must be noted that this 20 nt sequence has previously been reported to be resistant to RNAse E cleavage (41). As expected, northern blot analysis showed that the *acnB_+_*_20-3′UTR_-*lacZ* construct was sensitive to RyhB when cells were grown under Fe starvation (Figure 5D, lanes 5 and 6). This result could be explained by decreased binding affinity of apo-AcnB for *acnB_+_*_20-3′UTR_-*lacZ* construct. This possibility was tested by performing an AcnB_3xFLAG_ IP experiment using cells grown in Fe-poor medium. Data of IP experiments revealed *acnB* mRNA enrichment comparable to *acnB*_2749_ WT by apo-AcnB (Supplementary Figure S4C).

### Antagonist regulation of *acnB* mRNA and its effect on protein levels

**Figure 6.**
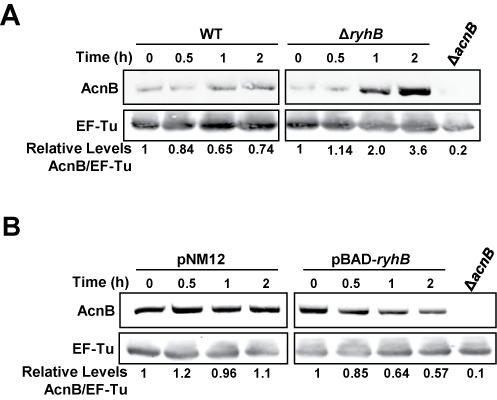
Both apo-AcnB stabilization and RyhB negative regulation affect AcnB levels. (**A**) Quantitative western blot of AcnB levels in Fe-depleted medium. Fe depletion was induced by addition of Dip (200 μM final) to WT (EM1055) or to Δ*ryhB* strains (EM1238) at time 0. Total proteins were extracted at the indicated time (h). Relative densitometry levels are indicated under the lanes. (**B**) Quantitative western blot of AcnB levels under Fe-rich conditions when RyhB sRNA was expressed in WT strain (EM1455) from pBAD-*ryhB* compared with an empty plasmid pNM12 (0.01% arabinose final). Proteins were extracted at the indicated time (h). EF-Tu protein was used as a loading control in both experiments. A polyclonal anti-AcnB antibody was used for hybridization and antibody specificity was determined using a protein extract from Δ*acnB* strain (strain JAB284 for panel A, JAB154 for panel B). Relative densitometry levels are indicated under the lanes. A IRDye 800CW-conjugated secondary antibody was used for quantiﬁcation.

To evaluate the effect RyhB on AcnB expression, we monitored AcnB levels by western blots and compared the results to native AcnB from WT and Δ*ryhB* cells grown under Fe starvation. Unexpectedly, AcnB levels remained stable over time when RyhB was induced in the presence of Dip (Figure 6A, left panel). In marked contrast, we observed a significant accumulation of AcnB (3.6-fold increase) in Δ*ryhB* cells 2 h after Dip treatment (Figure 6A, right panel). These results suggested that RyhB expression significantly repressed AcnB translation when cells grew under low Fe conditions.

We then investigated the effect of RyhB-induced repression of AcnB expression in Fe-rich LB medium (Figure 6B). Western blot analysis revealed an important decrease (43%) of AcnB levels after expression of RyhB sRNA from an arabinose-inducible BAD promoter in Δ*ryhB* cells (Figure 6B, right panel, 2 h). The decrease correlated with the repression observed on translational AcnB_2688_’-‘LacZ translational fusion (48% decrease) (Figure 4B, Ara). These results added support to the interpretation that RyhB directly blocked *acnB* mRNA translation initiation, thus significantly affect the levels of AcnB in these cells.

### Citrate interferes with *acnB* mRNA stabilization under low-iron conditions

**Figure 7.**
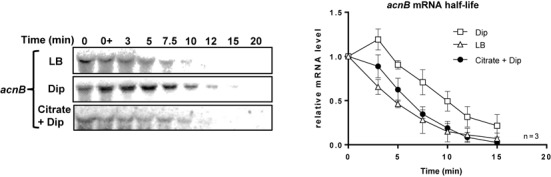
Addition of citrate, the AcnB substrate, abolishes *acnB* mRNA stabilization. Northern blot of *acnB* mRNA half-life and corresponding densitometric curves of *acnB* mRNA are shown. Cells (EM1238) were grown in LB medium to an OD_600_ of 0.5 at which point citrate (30 mM final) or Dip (200 μM final) were added at indicated time. Thereafter rifampicin (250 μg/ml) was added at time 0 and RNA extraction was performed at the indicated times. Data are representative of two independent experiments performed in triplicates.

Low intracellular iron conditions favor the switch of enzymatic (holo) AcnB to its RNA-binding (apo) conformation. We asked whether citrate, the substrate of AcnB, could interfere with this switch. To answer this question, EM1238 (Δ*ryhB*) cells were used to monitor the effect of apo-AcnB alone on *acnB* mRNA. Thus, cells were grown in iron-rich LB medium until mid-log phase, at which point Dip or a combination of Dip and citrate were added, followed by rifampicin to assess *acnB* mRNA stability. Densitometry analysis indicated that *acnB* mRNA half-life was slightly stabilized in the presence of Dip, which correlated with the switch of holo- to apo-AcnB RNA under low iron (Figure 7, top and middle panels). In contrast, the addition of citrate decreased the stability of *acnB* in a way similar to growth conditions in medium alone (Figure 7). These data were consistent with the interpretation that the presence of citrate prevented the switch of holo- to apo-AcnB even under low Fe conditions.

## DISCUSSION

**Figure 8.**
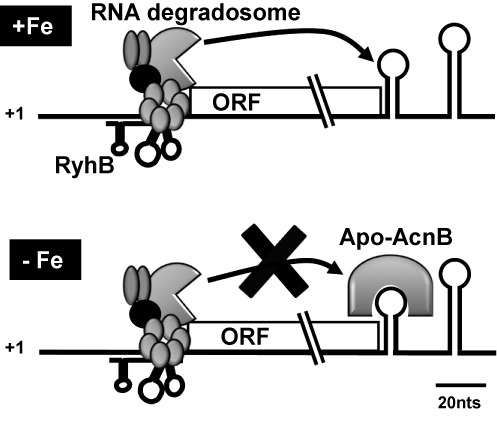
Working model of *acnB* mRNA double regulation by sRNA RyhB and RNA binding protein apo-AcnB (refer to the text for details).

A common outcome of sRNAs binding to their target mRNAs is rapid RNase E-mediated cleavage of the targets. However, the RyhB-induced *acnB* mRNA degradation that has been previously reported (15,31) can be prevented when cells are grown under low-iron conditions. Combined with previous studies, data presented here suggested that reduced intracellular iron promoted the switch of holo-AcnB to apo-AcnB with the resulting RNA-binding of *acnB* mRNA 3′UTR and protection against RyhB-induced mRNA degradation. According to the model proposed here, RyhB still binds to *acnB* RBS and partially blocks its translation initiation under low-iron conditions. However, our data support the interpretation that bound apo-AcnB seems to interfere with RNase E access to *acnB* mRNA cleavage site (Figure 8). Accordingly, this mechanism of regulation allows long-term expression homeostasis of AcnB as opposed to a significant stronger expression of AcnB in the absence of RyhB sRNA (Figure 6A). Although RyhB partially blocked *acnB* translation (Figures 4B and 6A, right), there was a steady production of AcnB (Figure 6A, left). This seemingly paradoxical observation may be explained by the functional nature of AcnB. When cells grow under iron starvation, most RyhB targets become degraded. These degraded targets encode single-function enzymes, whose activity would falter in the absence of sufficient amounts of iron as cofactor. In contrast, bifunctional AcnB switches functions despite low intracellular iron level. When it adopts its catalytic conformation, aconitase has an iron-dependent metabolic function that participates to the TCA cycle. As an RNA-binding protein, apo-AcnB regulates expression of various transcripts such as *acnB* and *ftsH* (3) despite environmental variations of iron concentrations and maintains steady expression of *acnB* that allows sufficient quantities of cellular AcnB to modulate both activities.

The question may be raised concerning the reason why two effectors, a sRNA and a RNA-binding protein, would regulate *acnB* mRNA that results, in any case, in similar expression levels of AcnB? Whereas these findings suggest a robust homeostatic system operating in *E. coli*, the presence of citrate reduced *acnB* stability under iron starvation conditions (Figure 7). These data are in agreement with previous observations indicating that citrate, the substrate of AcnB, prevents AcnB from switching to the apo-form (Varghese). This explains why citrate can reduce the expression of *acnB* even under conditions of low iron. It can be suggested that the sensitivity to citrate may allow the system to sense and respond to metabolic changes. This possibility is especially relevant as citrate can also act as an iron chelator, helping the cell to cope with low-iron conditions (42,43). Thus under growth conditions of low iron but high citrate, AcnB expression would be reduced (less apo-AcnB isoform) and iron acquisition would be increased in the presence of citrate. These growth conditions would favor activity of AcnB. In contrast, when both iron and citrate are low, there would be more AcnB in its apo-AcnB conformation, thus favoring its RNA-binding activity.

Our study suggests a sRNA-induced RNase E mRNA cleavage close to the 3′UTR of *acnB* mRNA, some 2600 nts from the sRNA binding site. Although we have previously shown a distal sRNA-induced RNase E cleavage on *sodB* mRNA 350 nt away from the sRNA binding site, this is a considerably longer distance. Such a distal location suggests the importance of regulating *acnB* mRNA stability accordingly to the RNA binding ability of AcnB. A similar system has been identified with transcripts regulated by microRNA (miRNA) in eukaryotes. In general, miRNAs are post-transcriptional repressors that act together with a protein complex named miRNA-induced-silencing-complex (miRISC). The multimeric complex binds imperfect complementary sequences of 3′UTR mRNA to repress translation. Frequently, RNA degradation follows miRNA pairing by stimulating deadenylation and decapping events (44–46). However, there are some examples where RNA binding proteins antagonize miRNA silencing. One of them occurs in human hepatocarcinoma cells where amino acids starvation leads to HuR protein oligomerization and binding to 3′UTR, impeding miRNA pairing (47,48). Here, we present data that support a similar model in which RyhB-induced degradation is prevented by the RNA binding protein apo-AcnB.

A key point about the mechanism of protection against sRNA-induced *acnB* cleavage is the close proximity of AcnB binding and RNase E cleavage sites. Evidence for this interpretation was obtained by increasing the distance between both sites by inserting a 20 nt sequence, previously shown to resist RNase E cleavage. Results indicated that increasing the distance abolished the apo-AcnB protection when RyhB was expressed (Figure 5D, *acnB*_+20-3′UTR_). This finding was similar to a report in which *rpsO* stop codon was distanced from a nearby RNase E cleavage site located in the mRNA 3′UTR and that resulted in reduced cleavage protection by ribosomes (41). In spite of these findings, the possibility remained that introduction of a 20 nt sequence in the 3′UTR of *acnB* could prevent apo-AcnB binding, thus allowing cleavage. However, this possibility was excluded by performing RNA-IP of apo-AcnB. Data showed that similar levels of *acnB*_+20-3′UTR_ and WT *acnB* RNA were observed (Supplementary Figure S4C), suggesting that apo-AcnB to *acnB* exerted steric hindrance to RNase E degrading activity.

Autoregulated proteins such as AcnB are common in bacterial housekeeping genes as observed in the case of many ribosomal proteins and tRNA regulators (49–52). For example, *rpmL-rplT* transcript is repressed when the second gene product, ribosomal protein L20, is overproduced. The binding of L20 protein to a pseudoknot located in *rpmL*-*rplT* 5′UTR blocks 30S ribosomal association with mRNA thereby preventing translation initiation (52,53). A similar case is ribosomal protein RpsO which binds structures in the 5′UTR of its own mRNA when it is overexpressed. Bound RpsO prevents translation initiation of *rpsO* mRNA, which becomes destabilized by exposed RNase E cleavage sites (41,54). These multiple levels of regulation/autoregulation of the same mRNA seem essential for highly regulated constitutive genes or global regulators.

Cellular adaptation to low iron growth remains a major stress for bacteria. When cells grow under low Fe conditions, many enzymes become virtually inactive in the absence of their iron cofactor. Under these stressful environmental conditions, these iron-dependent enzymes/proteins are repressed through sRNA-induced degradation of their mRNAs whereas in other cases some proteins/enzymes such as AcnB switch to a different functional form.

## SUPPLEMENTARY DATA

Supplementary Data are available at NAR Online.

SUPPLEMENTARY DATA
