# Supplementary material for: The iron-sensing aconitase B binds its own mRNA to prevent sRNA-induced mRNA cleavage
Source: Nucleic Acids Res. 2014 Aug 4;42(15):10023–36. doi: 10.1093/nar/gku649 (PMC4150767; doi:10.1093/nar/gku649)
Supplement: SUPPLEMENTARY DATA [file supp_42_15_10023__index.html]

The iron-sensing aconitase B binds its own mRNA to prevent sRNA-induced mRNA cleavage — The iron-sensing aconitase B binds its own mRNA to prevent sRNA-induced mRNA cleavage — SUPPLEMENTARY DATA 

# The iron-sensing aconitase B binds its own mRNA to prevent sRNA-induced mRNA cleavage

## SUPPLEMENTARY DATA

**Files in this Data Supplement:**

- SUPPLEMENTARY DATA
