# Supplementary material for: The iron-sensing aconitase B binds its own mRNA to prevent sRNA-induced mRNA cleavage
Source: Nucleic Acids Res. 2014 Aug 4;42(15):10023–36. doi: 10.1093/nar/gku649 (PMC4150767; doi:10.1093/nar/gku649)
Supplement: SUPPLEMENTARY DATA [file supp_gku649_nar-00950-z-2014-File010.pdf]

**Supplemental Material**

# The iron-sensing aconitase B binds its own mRNA to prevent sRNA-induced mRNA cleavage

Julie-Anna M. Benjamin and Eric Massé*

Department of Biochemistry, RNA Group, University of Sherbrooke, Sherbrooke, Québec, Canada.

*Corresponding author.
Department of Biochemistry
University of Sherbrooke
3201 Jean Mignault
Sherbrooke, Qc, J1E 4K8
Canada

Phone (819) 821-8000 ext. 75475
EMAIL: eric.masse@usherbrooke.ca;

**This File includes**

**Supplementary Figures S1 to S4**

**Supplemental Material and Methods**

**Supplementary Tables S1 to S3**

**Supplementary References**

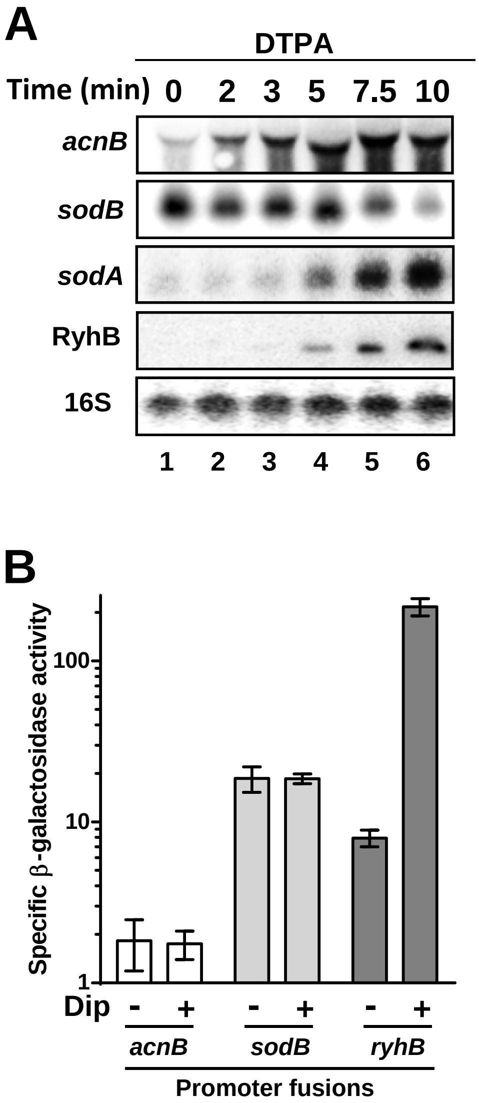


**Figure S1**. Effects of iron chelation on *acnB* expression. (A) Northern blot of total RNA hybridized with *acnB*, *sodB*, *sodA*, RyhB and 16S probes. RNA extraction was performed after addition of the iron chelator diethylene triamine pentaacetic acid (DTPA, final concentration of 100 μM) at time 0 to induce RyhB expression in EM1055 cells. Time course was performed at indicated time, at an $OD_{600}$ of 0.5. *sodB* mRNA was used as control for RyhB sRNA negative target and *sodA* mRNA as Fur protein negative target. 16S rRNA was used as a loading control. (B) Effect of Fe starvation on β-galactosidase activity of *acnB*, *sodB* and *ryhB* promoter transcriptional *lacZ* fusions in Δ*ryhB* strains (EM1238). Fe chelator Dip (200 μM) was added or not at an $OD_{600}$ of 0.1 and specific activity was measured in triplicate. Transcriptional *sodB-lacZ* fusion was used as a negative control and transcriptional *ryhB-lacZ* fusion as a positive control for Fe-dependent promoter regulation.

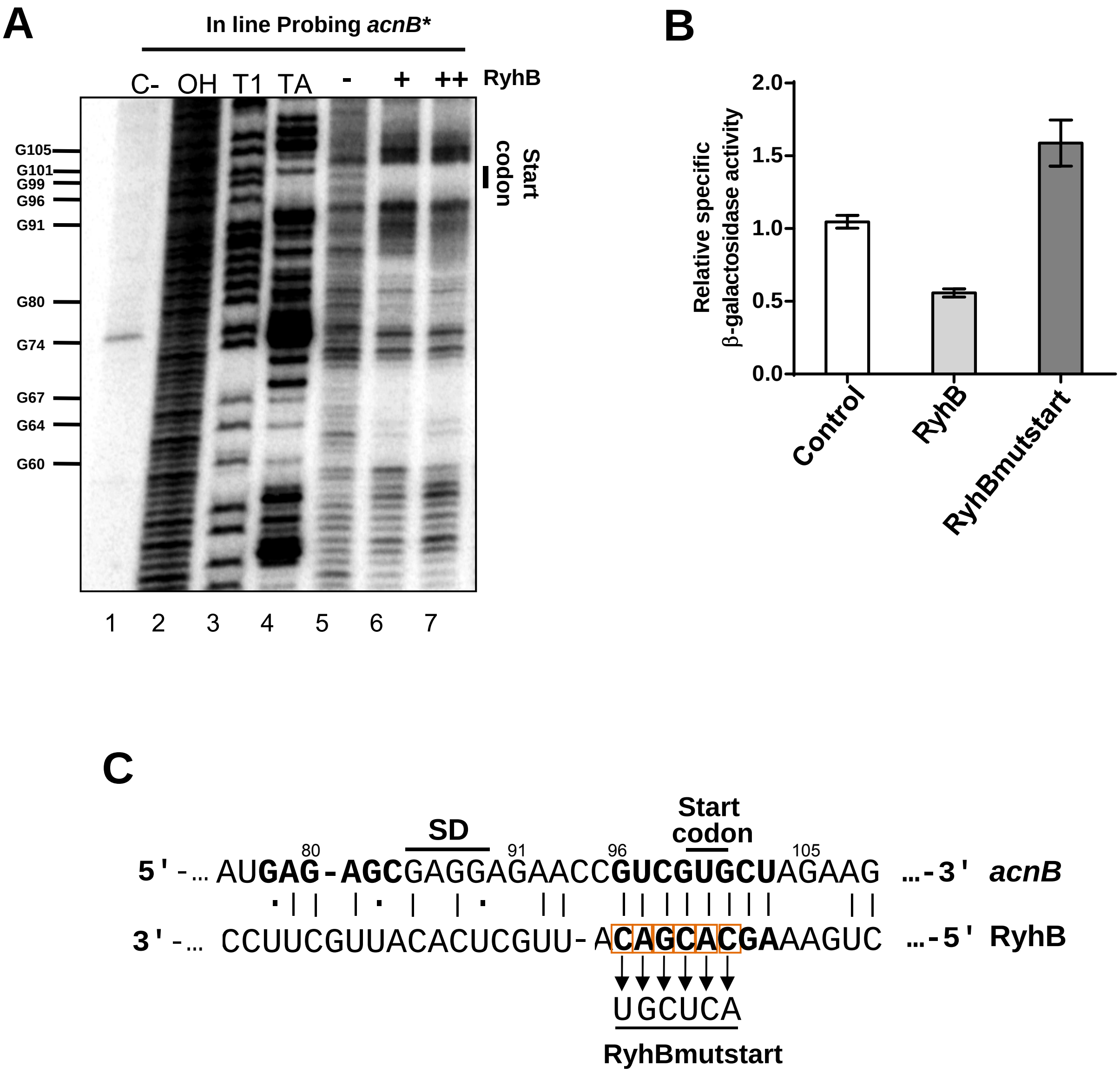


**Figure S2**. RyhB sRNA pairs to the translational initiation region of *acnB* mRNA. (A) *In vitro* pairing between sRNA RyhB and 5'end-radiolabelled *acnB* mRNA. (Lane 1) Control of *acnB* mRNA transcript alone. In-line probing reaction where *acnB* 5'UTR RNA transcript was incubated with RyhB sRNA in rates of 1:0, 1:10 and 1:20 (Lanes 5-7). (Lane 2) NaOH ladder. (Lane 3) RNase T1 ladder. (Lanes 4) RNase TA ladder. (B) The translational fusion $AcnB_{177}$-LacZ is specifically repressed by RyhB when induced from pBAD-*ryhB*. The sRNA RyhB is induced by Ara (0.01%) at an $OD_{600}$ of 0.1 and specific β-galactosidase activity has been measured 2h later. Expression of the RyhBmutstart (shown in (C)) from the pBAD-*ryhB*mutstart construct does not repress translation of $AcnB_{177}$-LacZ translational fusion. This absence of repression suggests that the 6 nt-long mutation in RyhBmutstart prevents pairing between RyhB and *acnB* mRNA. The empty vector pNM12 has been used as a control. Specific activity was measured in triplicate and standard deviation are presented on graph. (C) *In sillico* pairing prediction of RyhB sRNA on *acnB* mRNA on its translational initiation region by TargetRNA (57).

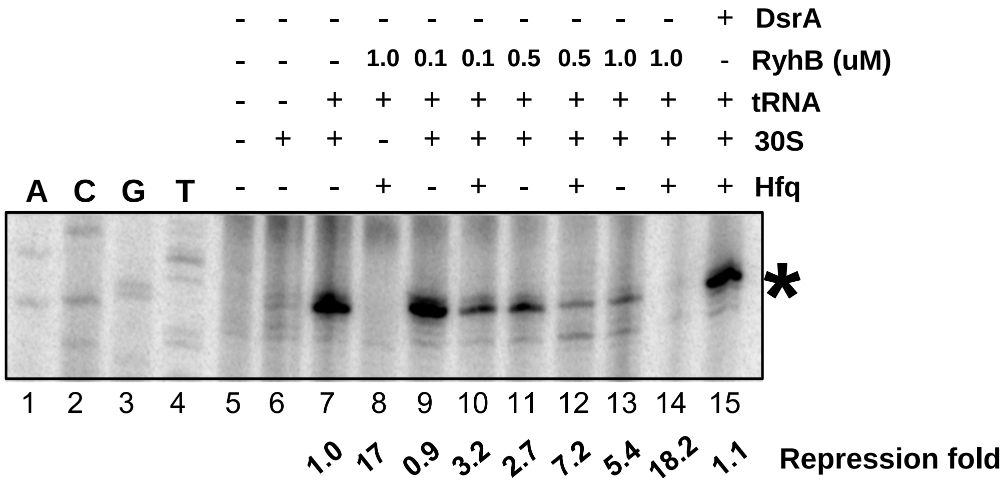


**Figure S3**. Toeprinting assays suggesting that RyhB sRNA and Hfq prevented 30S complex formation on *acnB* mRNA. Unlabeled *acnB* was incubated in the presence of increasing amount of RyhB (lanes 8 to 14) in the presence (lanes 8, 10, 12, 14) or absence (lanes 7, 9, 11, 13) of Hfq (0.5 μM). Ratio of *acnB*:Hfq:RyhB were 1:2.5:0.1 (lane 10), 1:2.5:0.5 (lane 12) and 1:2.5:1.0 (lane 14). ACGT refers to sequencing ladder generated with the same oligonucleotide (EM1484) that was used for toeprinting analysis. Toeprinting reverse transcription was performed with γ-$P^{32}$ 5'-end labelled oligonucleotide. Densitometry analysis of fold of repression relative to toeprint control (lane 7) is shown. DsrA sRNA was used as a negative control of translational repression of *acnB* mRNA (lane 15).

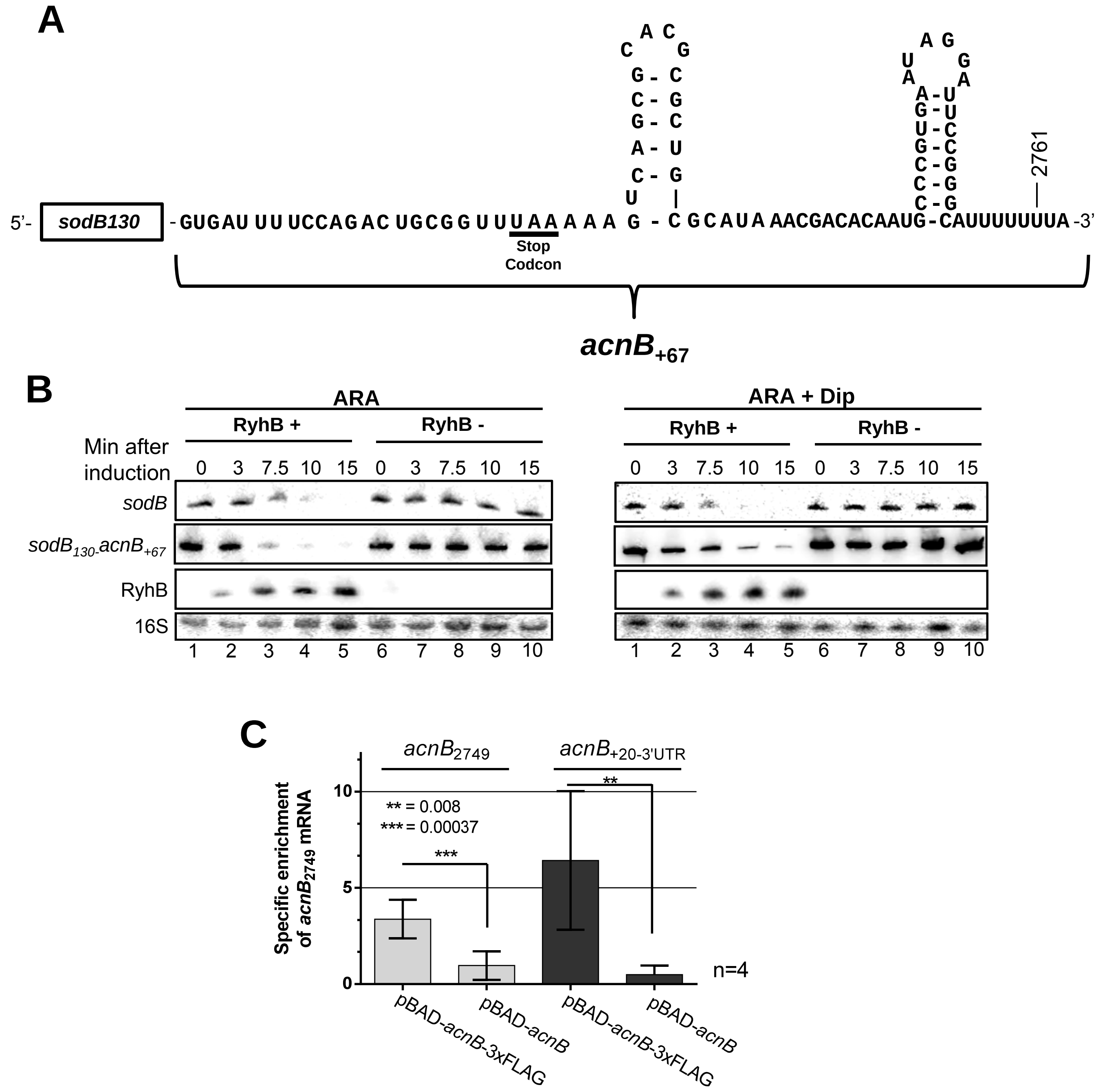


**Figure S4**

The *acnB* 3'UTR acts ectopically to stabilize a $sodB_{130}$-$acnB_{+67}$ chimeric fusion. (A) Schematic view of $sodB_{130}$-$acnB_{+67}$ construct in which *sodB* 5'UTR RNA (from nt 1 to nt 341) was fused to *acnB* from nt 2670 to nt 2761. (B) Northern blots of total RNA using an RNA probe specific to *sodB* showing the effect of RyhB expression on $sodB_{130}$-$acnB_{+67}$ construct. Left panel: arabinose (0.05% final) was added to induce expression of RyhB (pBAD-*ryhB*) or the control (pNM12) at an $OD_{600}$ of 0.5. Right panel, same as left panel except the Fe chelator Dip (200 µM final) was added 10 min before RyhB induction. Expression of endogenous *sodB* mRNA and RyhB sRNA is also shown. 16S ribosomal is used as a control. (C) $AcnB_{3xFLAG}$ RNA-IP experiment of $acnB_{2749}$ construct was compared with $acnB_{2749\text{-}+20}$ construct. In both construct, *acnB* were expressed from endogenous promoter in Fe-poor media. Then, AcnB was expressed (JAB292) in cells with plasmids pBAD-$acnB_{3xFLAG}$ and pBAD-*acnB*. RNA-IP was performed at an $OD_{600}$ of 0.5. qRT-PCR was performed for determination of $acnB_{2749}$ and $acnB_{2749\text{-}+20}$ specific enrichment by using 3'-end specific probes. Mean and s.d. of experiments are shown.

## Suplementary Material and Methods

*Strain and plasmids*

Derivatives of *E. coli* MG1655 were used in all experiments. DH5α strain was used for routine cloning procedures. Construction of *acnB*$_{117}$ *–lacZ, sodB-lacZ* and *ryhB-lacZ* (EM487-EM488) transcriptional fusions was constructed as described in Mitarai *et al.* (1).

To construct the AcnB$_{2688}$-LacZ translational fusion, a PCR product obtained with oligonucleotides EM531 and EM549 was digested with *Bam*HI and ligated into *Bam*HI-digested pRS1551 (creating an in-frame translational fusion with *lacZ*). This fusion was then delivered as a single copy into the chromosome of different strains (see table S1) at the λ *attI* site, as described previously (2). Stable lysogens were screened for single insertion of recombinant λ by PCR (3).

To construct the *acnB*$_{2749}$*–lacZ* transcriptional fusion, a PCR product was generated with oligonucleotides EM531 and EM1557 and then digested with *Bam*H1 and ligated into *Bam*H1-digested pFRΔ vector. To generate mutated versions of *acnB*$_{2749}$*–lacZ*, a PCR reaction was performed using *acnB*$_{2749}$ PCR product as the DNA template with the following oligonucleotides: *acnB*$_{2749\text{-Mstem}}$ (EM531-EM1956), *acnB*$_{2749\text{-stem}}$ (EM531-EM2004) and *acnB*$_{\text{CC-stop-GCC}}$ (EM531-EM1821). For *acnB*$_{+20\text{-3'UTR}}$ construct, a two-step PCR strategy was used. While the first PCR was performed with oligonucleotides EM531-EM1958, the second step was performed by using oligonucleotides EM531-EM1959. The *acnB*$_{+20\text{-GCC}}$ construct was obtained by performing a PCR reaction with oligonucleotides EM531-EM2057 followed by another amplification reaction with oligonucleotides EM531 and EM2058. The resulting PCR product was then digested with *Bam*HI and ligated into *Bam*HI-digested pFRΔ. The cloning strategy used to generate plasmids pBAD33-*acnB*$_{2749}$, pBAD33-*acnB*$_{2749\text{Mstem}}$, pBAD33-*acnB*$_{2749\text{-stem}}$ and pBAD33-*acnB*$_{+20\text{-3'UTR}}$ was the same as described above for pFRΔ cloning except that the vector used for ligation was *Bam*H1-digested pBAD33. Those plasmids were transformed into appropriate strains before performing experiments.

To construct *sodB*$_{130}$-*acnB*$_{3'}$, a three-step PCR strategy was performed. A first PCR product (*sodB*$_{130}$) was obtained by using oligonucleotides EM423-EM424 and *E. coli* genomic DNA. Then a second PCR was performed with oligonucleotides EM423-EM1957 on the previous *sodB*$_{130}$ PCR product as template. A third PCR product was obtained by amplifying the previous PCR product with EM423 and EM1939. Finally, the PCR product was digested with *Eco*R1 and *Bam*HI and then ligated into *Eco*RI/*Bam*H1-digested pFRΔ. The resulting transcriptional fusion was delivered as a single copy into the chromosome as described previously.

The plasmid pBAD24-*acnB* was generated by PCR reactions using oligonucleotides EM1695-EM1258 to produce a promoter-less *acnB* fragment. The resulting PCR product was digested with *Bam*H1/*Pst*1 and ligated into *Bam*H1/*Pst*1-digested pBAD24. To construct the pBAD24-*acnB-3x-FLAG* plasmid, the following

oligonucleotides (EM1689-EM1747 and EM1695-EM549) were used to generate two PCR products. Following this, both products were mixed and amplified with oligonucleotides EM1695-EM1748 to generate *acnB-3x-FLAG*. Then, the final PCR was digested with *Bam*H1 and ligated into *Bam*H1-digested pBAD24.

Plasmid pET21b-*acnB-FLAG* was produced by a two-step PCR reaction to generate a promoter-less *acnB* fragment using oligonucleotides EM1259-EM1260, which then served as template for a second PCR reaction with oligonucleotides EM1259-EM1261. The final PCR product was digested with *Nde*1/*Xho*1 and ligated into pET21b.

Plasmid pGD3 is a pBAD33 derivative (4) that has been mutagenized to insert a *Xho*I site at the -6 to -1 region of the +1 transcription start site (see (5) for details). pGD3-*ryhB* was generated by a PCR reaction producing a promoter-less *ryhB* DNA fragment with a *Xho*I site upstream and an *Eco*RI site downstream (oligonucleotides EM1572-EM455). The PCR product was digested with *Xho*I and *Eco*RI and ligated into *Xho*I/*Eco*RI-digested pGD3.

*RNA secondary structure probing*

In line probing was performed as described previously (6). γ-5'-$^{32}$P-end labelled *acnB*$_{177}$ RNA (final concentration of 0.1 µM) was incubated with 1 µM or 2 µM RyhB RNA. The *acnB*$_{177}$ and RyhB RNA were produced using T7 RNA polymerase from a PCR product (see Table S3 for oligonucleotides). Ribonucleases T1 (0.1 U) (Ambion) and TA (2.5 U) (Jena Biosciences) were used for 5 min at 37$^{o}$C in sequencing buffer (Ambion). Alkaline ladder was generated in alkaline buffer (Ambion) for 5 min at 90$^{o}$C.

*Toeprinting assays*

Toeprinting analysis and 30S purification has been performed as described previously (5,7). Purification of Hfq protein was performed as described previously (8).

**Table S1**

List of all the strains and plasmids used in this study.

| **Strain Number** | **Relevant Markers** | **Reference/Source** |
|---|---|---|
| EM1055 | MG1655 Δ*lacX*74 | (9) |
| EM1238 | EM1055 Δ*ryhB*::*cat* | (9) |
| JF133 | EM1055 *rne131 zce*-726::Tn*10*, *ryhB*::*cat* | (10) |
| EM1451 | EM1055 Δ*ara*714 *leu*$^+$ | (11) |
| EM1455 | EM1055 Δ*ara*714 *leu*$^+$ *ryhB*::*cat* | EM1451 + P1vir EM1238 |
| DY330 | W3110 Δl*acU169 gal490* ΔcI857 Δ (*cro-bioA*) | (12) |
| GD363 | DY330 + *ryhB*::*tet* cassette | (5) |
| KP1135 | EM1055 Δ*ara*714 *leu*$^+$ *ryhB*::*tet* | EM1451 + P1vir GD363 |
| JF222 | EM1055 *ryhB*::*cat* λ*acnB*$_{117}$'-*lacZ* | EM1238 + λpFRΔ-*acnB*$_{117}$'-lacZ |

| | | |
|---|---|---|
| JAB017 | EM1055 λ*ryhB*'-*lacZ* | EM1055 + λpFRΔ-*ryhB*'-lacZ |
| KP519 | EM1055 $sodB_{130}$'-*lacZ ryhB*::*cat* | (10) |
| JAB242 | EM1055 $sodB_{130}$-$acnB_{+67}$ | EM1055 + λpFRΔ-$sodB_{130}$-$acnB_{3'}$-*lacZ* |
| JAB243 | EM1055 *ryhB*::*cat* $sodB_{130}$-$acnB_{+67}$ | EM1238 + λpFRΔ-$sodB_{130}$-$acnB_{3'}$-*lacZ* |
| JAB246 | KP1135 Δ*ara*714 $leu^+$ *ryhB*::*tet* $sodB_{130}$-$acnB_{+67}$ | KP1135 + λpFRΔ-$sodB_{130}$-$acnB_{3'}$-*lacZ* |
| GD432 | JW0014-5 (*acnB*736::*kan*) | (Keio collection, Yale) |
| JAB154 | EM1055 Δ*ara*714 $leu^+$ *acnB*736::*kan* | EM1451 + P1vir GD432 |
| JAB212 | EM1055 Δ*ara*714 $leu^+$ *ryhB*::*tet acnB*736::*kan* | JAB154 + P1vir GD363 |
| JAB284 | EM1055 *acnB*736::*kan* | EM1055 + P1vir GD432 |
| JAB292 | EM1055 *ryhB*::*tet acnB*736::*kan* | JAB284 + P1vir GD363 |
| JAB153 | EM1055 Δ*ara*714 $leu^+$ *ryhB*::*cat acnB*736::*kan* | EM1455 + P1vir GD432 |
| JAB051 | EM1055 Δ*ara*714 $leu^+$ *ryhB*::*cat* λ$AcnB_{2688}$'-'LacZ | EM1055 + λpRS1551 $AcnB_{2688}$'-'LacZ |
| JAB068 | EM1055 *ryhB*::*cat* λ $AcnB_{2688}$'-'LacZ | EM1238 + λpRS1551-$AcnB_{2688}$'-'LacZ |
| KP760 | EM1055 Δ*ara*714 $leu^+$ *ryhB*::*cat* $AcnB_{2688}$'-'LacZ | EM1455 + λpRS1551-$AcnB_{2688}$'-'LacZ |
| **Plasmids** | **Description** | **Reference/Sources** |
| pBAD24 | | (4) |
| pNM12 | pBAD24 derivative | (13) |
| pBAD-*ryhB* | pNM12 + RyhB (arabinose-inducible promoter) | (14) |
| pBAD-*ryhB*mutstart | pNM12 + RyhBmutstart (arabinose-inducible promoter) | This study |
| pGD3 | pBAD33 derivative | (5) |
| pGD3-*ryhB* | pGD3 + RyhB (arabinose-inducible promoter) | This study |
| pBAD24-*acnB*-*3xFlag* | pBAD24 + *acnB* + 3xFlag tag (arabinose inducible promoter) | This study |
| pBAD24-*acnB* | pBAD24 + *acnB* (arabinose inducible promoter) | This study |
| pRS1551 | Plasmid for construction of translational fusions | (2) |
| pFRΔ | pRS1553 derivative (for transcriptional fusions) | (15) |
| pFRΔ-$acnB_{2688}$ | pFRΔ + $acnB_{2688}$ | This study |
| pFRΔ-$acnB_{2749}$ | pFRΔ + $acnB_{2749}$ | This study |
| pFRΔ-$acnB_{2749\text{-Mstem}}$ | pFRΔ + $acnB_{2749\text{-Mstem}}$ | This study |
| pFRΔ-$acnB_{2749\text{-stem}}$ | pFRΔ + $acnB_{2749\text{-stem}}$ | This study |
| pFRΔ-$acnB_{\text{CC-stop-GCC}}$ | pFRΔ + $acnB_{\text{CC-stop-GCC}}$ | This study |
| pFRΔ-$acnB_{+20\text{-3'UTR}}$ | pFRΔ + $acnB_{+20\text{-3'UTR}}$ | This study |
| pFRΔ-$acnB_{+20\text{-GCC}}$ | pFRΔ + $acnB_{+20\text{-GCC}}$ | This study |
| pFRΔ-$sodB_{130}$-$acnB_{3'}$ | pFRΔ + $sodB_{130}$-$acnB_{3'}$ | This study |
| pBAD33 | | (4) |
| pBAD33- | pBAD33 + $acnB_{2749}$ (arabinose inducible promoter) | This study |

| | | |
|---|---|---|
| *acnB*$_{2749}$ | | |
| pBAD33-*acnB*$_{2749\text{-Mstem}}$ | pBAD33 + *acnB*$_{2749\text{-Mstem}}$ (arabinose inducible promoter) | This study |
| pBAD33-*acnB*$_{2749\text{-stem}}$ | pBAD33 + *acnB*$_{2749\text{stem}}$ (arabinose inducible promoter) | This study |
| pBAD33-*acnB*$_{+20\text{-}3\text{'UTR}}$ | pBAD33 + *acnB*$_{+20\text{-}3\text{'UTR}}$ (arabinose inducible promoter) | This study |

**Table S2**

List of all the oligonucleotides used in this study.

| **Oligo number** | **Sequence 5'-3'** |
|---|---|
| EM88 | TGTAATACGACTCACTATAGGGCGATCAGGAAGACCCTCGC |
| EM89 | AAAAGCCAGCACCCGGCTGGC |
| EM125 | TGTAATACGACTCACTATAGGTGCGGTACTGGGCATTTACCC |
| EM140 | TAATACGACTCACTATAGGGAGAGCATCACCAGGTCAGCCGAG |
| EM141 | GATGAAGTGCGTGCTGGTGGC |
| EM188 | TAATACGACTCACTATAGGGAGACCAGGCAGTTCCAGTAGAAAG |
| EM189 | GCTAAAGATGCTCTGGCACCG |
| EM247 | GTAACTTCCCGAACCGTCTG |
| EM293 | TAATACGACTCACTATAGGGAGACGCTTTACGCCCAGTAATTCC |
| EM294 | CTCCTACGGGAGGCAGCAGT |
| EM305 | TAATACGACTCACTATAGGGAGAGCTTTGAAGTTATCAACGGAGC |
| EM306 | TCAGACCTACGTAAACAACGC |
| EM411 | ACATCCGTACTCTTCGTGCG |
| EM412 | TGTAATACGACTCACTATAGGAGTCCAGGTTTTAGTTTCGCC |
| EM423 | CAGTTGAATTCGGCGTGTATGTCGGCAACGG |
| EM424 | GCTAGGGATCCTCGATGGTTTCCGCAGAAATG |
| EM455 | CAGGCTGAAAATCTTCTCTCATC |
| EM487 | CAGTTGAATTCCCGTGTTTCTGCGTGGCG |
| EM488 | GCTAGGGATCCGCGAGACAATAATAATCATTC |
| EM531 | GCTAGGGATCCTAGACCATCCTTAACGATTCAG |
| EM549 | GCTAGGGATCCGCAGGTCTGGAAAATCACCC |
| EM635 | TGTAATACGACTCACTATAGAACACATCAGATTTCCTGGTG |
| EM636 | AAATCCCGACCCTGAGGG |
| EM1258 | CCAATGCATTGGTTCTGCAGTTAAACCGCAGTCTGGAAAATCAC |
| EM1259 | GGAATTCCATATG TGCGGTACTGGGCATTTACCC |
| EM1260 | AACCGCAGTCTGGAAAATCACC |
| EM1261 | CCGCTCGAGTTATTTATCGTCATCGTCTTTGTAGTC AACCGCAGTCTGGAAAATCACC |
| EM1484 | GGTTTGCATCCAGGGGTTTGG |
| EM1557 | GCTAGGGATCCGAATCTCATTCACCGGGCATTG |
| EM1572 | GGGGCTCGAGGCGATCAGGAAGACCCTCGC |
| EM1573 | GAATCTCATTCACCGGGCATTG |
| EM1578 | TGTAATACGACTCACTATAGGAGTACCAGACCTACGTGGCG |
| EM1596 | GAAATAAAAAAATGC CCG GGA ATC |
| EM1689 | GACTACAAAGACCATGACGGTGATTATAAAGATCATGATATCGACTA CAAAGATGACGACGATAAATAGTAAGTGTAGGCTGGAGCTGCT |
| EM1695 | GCTAGGGATCCTGCGGTACTGGGCATTTACCC |

| | |
|---|---|
| EM1744 | GCTAGGGATCCTGCGGTACTGGGCATTTACCC |
| EM1747 | CGCAGTCTGGAAAATCACCCGACTACAAAGACCATGACGGTG |
| EM1748 | GCTAGGGATCCTTACTATTTATCGTCGTCATCTTTG |
| EM1767 | CTCTACAAGTGCGGCCATTTGGTT |
| EM1821 | GCTAGGGATCCGGCATTGTGTCGTTTATGCGCAGCGCGTGCGCTGACGGCTTAGGCCGCAGTCTGGAAAATCAC |
| EM1939 | GCTAGGGATCCGTGATTTTCCAGACTGCGGTTTAAAAAGTCAGCGCACGCGCTGCGCATAAACGACACAATGCCCGGTGAATGAGATTCCCGGGCATTTTTTTATTTCGAATTCAGTCAG |
| EM1956 | GCTAGGGATCCGAATCTCATTCACCGGGCATTGTGTCGTTTATGCGGTGCGCGTGCGCTGACTTTTTAAACCGCAG |
| EM1957 | CCGCAGTCTGGAAAATCACCTCGATGGTTTCCGCAGAAATGTG |
| EM1958 | GTGATTTTCCAGACTGCGGTTTAAAAAGCGUCGCUAAUUCUUGCCAGGTCAGCGCACGCGCTGCG |
| EM1959 | GCTAGGGATCCGAAATAAAAAAATGCCCGGGAATC |
| EM2004 | GCTAGGGATCCGAATCTCATTCACCGGGCATTGTGTCGTTTATGCGGTGCGCGTGCGCACACTTTTTAAACCGCAG |
| EM2057 | CTGGCAAGAATTAGCGACGCGGCTTAGGCCGCAGTCTGGAAAATC |
| EM2058 | GCTAGGGATCCGGAATCTCATTCACCGGGCATTGTGTCGTTTATGCGCAGCGCGTGCGCTGACCTGGCAAGAATTAGCGACGC |

**Table S3**

A list of all the RNA transcripts produced by T7 RNA polymerase in vitro transcription. Indicated are the final product (and its purpose), the oligonucleotides used to generate the DNA template by PCR and the DNA template used for the PCR.

| **Final product** | **Oligonucleotides** | **DNA template** |
|---|---|---|
| *acnB* (Northern probe) | EM140 - EM141 | Genomic DNA |
| RyhB (Northern probe) | EM88 - EM89 | Genomic DNA |
| *sodB* (Northern probe) | EM188 - EM189 | Genomic DNA |
| *sodA* (Northern probe) | EM305 - EM306 | Genomic DNA |
| *hns* (Northern probe) | EM411 - EM412 | Genomic DNA |
| 16S rRNA (Northern probe) | EM293 - EM294 | Genomic DNA |
| *acnB*$_{177}$ (5’end) (PbAc probing/toeprinting) | EM125 – EM1767 | Genomic DNA |
| *acnB* (3’end) (Footprint/ degradation assay) | EM1578 – EM1596 | Genomic DNA or pFRΔ-*acnB*$_{CC\text{-}stop\text{-}GCC}$ |
| RyhB (PbAc probing/toeprinting) | EM88-EM89 | Genomic DNA |
| DsrA (PbAc probing/toeprinting) | EM635-EM636 | Genomic DNA |
| *acnB* (qRT-PCR) | EM247-EM1573 | Genomic DNA |

**References**

1. Mitarai, N., Benjamin, J.A., Krishna, S., Semsey, S., Csiszovszki, Z., Masse, E. and Sneppen, K. (2009) Dynamic features of gene expression control by small regulatory RNAs. *Proceedings of the National Academy of Sciences of the United States of America*, **106**, 10655-10659.
2. Simons, R., Houman, F. and Kleckner, N. (1987) Improved single and multicopy lac-based cloning vectors for protein and operon fusions. *Gene*, **53**, 85-96.

3. Powell, B.S., Rivas, M., Court, D., Nakamura, Y. and Turnbough Jr, C. (1994) Rapid confirmation of single copy lambda prophage integration by PCR. *Nucleic acids research*, **22**, 5765.
4. Guzman, L.-M., Belin, D., Carson, M.J. and Beckwith, J. (1995) Tight regulation, modulation, and high-level expression by vectors containing the arabinose PBAD promoter. *Journal of bacteriology*, **177**, 4121-4130.
5. Desnoyers, G. and Massé, E. (2012) Noncanonical repression of translation initiation through small RNA recruitment of the RNA chaperone Hfq. *Genes & development*, **26**, 726-739.
6. Regulski, E.E. and Breaker, R.R. (2008), *Post-Transcriptional Gene Regulation*. Springer, pp. 53-67.
7. Morita, M.T., Tanaka, Y., Kodama, T.S., Kyogoku, Y., Yanagi, H. and Yura, T. (1999) Translational induction of heat shock transcription factor ς32: evidence for a built-in RNA thermosensor. *Genes & development*, **13**, 655-665.
8. Prevost, K., Salvail, H., Desnoyers, G., Jacques, J.F., Phaneuf, E. and Masse, E. (2007) The small RNA RyhB activates the translation of shiA mRNA encoding a permease of shikimate, a compound involved in siderophore synthesis. *Molecular microbiology*, **64**, 1260-1273.
9. Masse, E. and Gottesman, S. (2002) A small RNA regulates the expression of genes involved in iron metabolism in Escherichia coli. *Proceedings of the National Academy of Sciences of the United States of America*, **99**, 4620-4625.
10. Prevost, K., Desnoyers, G., Jacques, J.F., Lavoie, F. and Masse, E. (2011) Small RNA-induced mRNA degradation achieved through both translation block and activated cleavage. *Genes & development*, **25**, 385-396.
11. Desnoyers, G., Morissette, A., Prevost, K. and Masse, E. (2009) Small RNA-induced differential degradation of the polycistronic mRNA iscRSUA. *The EMBO journal*, **28**, 1551-1561.
12. Yu, D., Ellis, H.M., Lee, E.-C., Jenkins, N.A. and Copeland, N.G. (2000) An efficient recombination system for chromosome engineering in Escherichia coli. *Proceedings of the National Academy of Sciences*, **97**, 5978-5983.
13. Majdalani, N., Cunning, C., Sledjeski, D., Elliott, T. and Gottesman, S. (1998) DsrA RNA regulates translation of RpoS message by an anti-antisense mechanism, independent of its action as an antisilencer of transcription. *Proceedings of the National Academy of Sciences*, **95**, 12462-12467.
14. Masse, E., Escorcia, F.E. and Gottesman, S. (2003) Coupled degradation of a small regulatory RNA and its mRNA targets in Escherichia coli. *Genes & development*, **17**, 2374-2383.
15. Repoila, F. and Gottesman, S. (2001) Signal transduction cascade for regulation of RpoS: temperature regulation of DsrA. *Journal of bacteriology*, **183**, 4012-4023.
